# Discovery and Functional Evaluation of Antimicrobials

**DOI:** 10.3390/antibiotics10070765

**Published:** 2021-06-23

**Authors:** Kandasamy Saravanakumar, Kumar Vishven Naveen, Myeong-Hyeon Wang

**Affiliations:** Department of Bio-Health Convergence, Kangwon National University, Chuncheon 200-701, Korea; naveen.vishven@gmail.com

Microbial infections pose a continuous threat to human health and healthcare systems around the globe. The emergence of antibiotic-resistant microbes has been confronted as a serious challenge in bio-health therapeutics. Multidrug-resistant (MDR) pathogens are the reason for 700,000 annual deaths worldwide, and are expected to account for more than 10 million annual deaths by 2050. The available treatments and health services are seen in the predicaments involved in tackling the recent COVID-19 pandemic and its mutated viral strains. Additionally, these challenges have delimitated current therapeutic strategies. However, advancements in technology such as nanobiotechnology, immunoinformatics, antimicrobial peptides, the rediscovery of natural compounds, etc. have contrived to fight microbial threats. Therefore, this Special Issue focused on the ‘Discovery and Functional Evaluation of Antimicrobials’ in order to understand the microbes associated with health challenges and hunt for potential functional antimicrobials.

In this Special Issue, J. M. Ambrose and colleagues study the spike proteins associated with the SARS-CoV-2 virus and collate their immunogenic profiles. The study suggests the increased T-cell epitope antigenicity and moderate to high immunogenic potential for the best epitope/HLA combinations in current strains (USA, British, India, and South Africa) as compared to the Wuhan strain. In addition, the new variants’ associated peptides are predicted in the study, which can be utilized in vaccine development [1].

A wound is a condition of the compromised first line of defense and offers an easy penetration of microbes inside the body. Together with the pathophysiological environment, microbial infections curtail the wound healing process. This complication is more chronic in diabetes-associated conditions. In this regard, A. Sathiyaseelan and collaborators synthesized a multi-functional iron oxide nanoparticle (FeO NPs), employing *Pinus densiflora* extracts, and disclosed the detailed characterization and bioactivities. The study reported an FeO fabricated chitosan/poly (vinyl alcohol) nanocomposite sponge (CS/PVA-PD-FeO NCs) containing excellent wound healing and antimicrobial properties [2].

Similarly, another key study from this issue presents zinc oxide, titania, and silver nanocomposites (ZnO/TiO2/Ag-NCs) fabricated by fruit extracts of *Morinda citrifolia*. P. S. Mohan and colleagues report on the antibacterial effects of the ternary nanocomposite that involve disrupting the cell membrane of Gram-positive and Gram-negative bacteria [3].

Finally, research by M. Rubab and D.H. Oh compared the virulence factors and multidrug resistance features of Shiga toxin-producing *E. coli* (STEC). In the study, serotype O26 strains were reported as carrying the maximum number of virulence-associated factors [4].

Moreover, the presented Special Issue covers the works involved in the management of pathogenic and resistant bacterial strains. One study suggests the possible immunoinformatics approach to address SARS-CoV-2. Therefore, we do anticipate that the pathologist and clinicians working in the field will benefit from this Special Issue. However, additional special consideration is needed to elucidate the exact molecular mechanisms of newly identified antimicrobials to attain the stipulated hypothesis of the present issue.
